# Cognitive aging on latent constructs for visual processing capacity: a novel structural equation modeling framework with causal assumptions based on a theory of visual attention

**DOI:** 10.3389/fpsyg.2014.01596

**Published:** 2015-01-15

**Authors:** Simon Nielsen, L. Inge Wilms

**Affiliations:** Brain Rehabilitation Advanced Technology and Learning Laboratory, Department of Psychology, University of CopenhagenCopenhagen, Denmark

**Keywords:** cognitive aging, visual attention, visual short-term memory, structural equation modeling, a theory of visual attention, SEM, TVA

## Abstract

We examined the effects of normal aging on visual cognition in a sample of 112 healthy adults aged 60–75. A testbattery was designed to capture high-level measures of visual working memory and low-level measures of visuospatial attention and memory. To answer questions of how cognitive aging affects specific aspects of visual processing capacity, we used confirmatory factor analyses in Structural Equation Modeling (SEM; Model 2), informed by functional structures that were modeled with path analyses in SEM (Model 1). The results show that aging effects were selective to measures of visual processing speed compared to visual short-term memory (VSTM) capacity (Model 2). These results are consistent with some studies reporting selective aging effects on processing speed, and inconsistent with other studies reporting aging effects on both processing speed and VSTM capacity. In the discussion we argue that this discrepancy may be mediated by differences in age ranges, and variables of demography. The study demonstrates that SEM is a sensitive method to detect cognitive aging effects even within a narrow age-range, and a useful approach to structure the relationships between measured variables, and the cognitive functional foundation they supposedly represent.

## Introduction

The increased lifespan in the general population has also increased the risk of cognitive decline. This has emphasized the need for the development of methods to detect, delineate and remedy cognitive decline, which are easy to administer and sensitive to age-related changes. In the current study, we are particularly interested in how age affects different aspects of visual processing capacity, such as the encoding/processing speed into visual short-term memory (VSTM) and the capacity of VSTM. We apply a novel computerized test battery to capture behavioral measures of visuospatial attention and memory in 112 healthy adults between 60 and 75 years of age. The testbattery is designed to assess cognition at different levels of functional complexity and specificity, to provide a detailed insight into the cognitive variables affected by age. To analyse the relationship between age and cognition we use structural equation modeling (SEM), which is a powerful approach to model the structure between measured and latent variables. To enforce the integrity of the SEM models in describing actual cognitive constructs, we apply A Theory of Visual Attention (TVA; Bundesen, 1990) to make the causal assumptions, that the TVA parameters C and K represents fundamental measures of processing speed (C) and VSTM capacity (K). This allows us to test whether specific measures affected by age relate mostly to processing speed or VSTM capacity, or conversely whether processing speed or VSTM capacity is mostly affected by age. Two SEM models are presented in the study. Model 1 is a path analysis SEM that tests a hypothetical organization of the measured variables according to functional complexity (level of assessment) and specificity (relative dependency on processing speed vs. VSTM capacity). Model 2 is a confirmatory factor analysis SEM that examines how age influences latent constructs for processing speed and VSTM capacity, when these are derived from multiple distinct measures informed by Model 1.

### Background

#### Cognitive aging

Cognitive aging has been related to decline in several higher-order visual working memory (VWM) abilities such as, speed of reading (Connelly et al., 1991; Hartley et al., 1994) mental image manipulation (Berg et al., 1982; Dror and Kosslyn, 1994), and memory recall (Berg et al., 1982; Dror and Kosslyn, 1994; Anderson et al., 1998). Also, there is a general consensus between behavioral and neuroimaging studies that decline in task switching abilities affects VWM performance in old age due to lack of attentional control in the wake of distraction (West, 1999; Clapp et al., 2010; Anguera et al., 2013). This has been proposed to be caused by a general selective attention impairment pertaining to inhibition of task-irrelevant information (Gazzaley et al., 2005; Clapp and Gazzaley, 2012).

In addition, a number of studies have reported age related decline in more general cognitive mechanisms of processing capacity such as a reduction in visual short term memory (VSTM) capacity (Habekost et al., 2012; McAvinue et al., 2012), and in perceptual processing/encoding speed (Salthouse, 1996, 2000; Habekost et al., 2012; McAvinue et al., 2012), which may contribute to the impaired VWM performance in old age (Brown et al., 2012; Franceschini et al., 2012).

The range and diversity of observed impairment raise the question whether the decline may share a common ground being related to either decline in capacity or encoding/decoding into different stages of working memory.

To examine this further, we create two SEM models to determine the dependency between measures and to test a hypothesized hierarchical relation between several behavioral measures that are typically sensitive to age related changes. We do this using data obtained from a novel test battery that comprise fundamental visuospatial measures (processing speed, perceptual threshold, VSTM capacity) as well as intermediate (delayed recognition, attention span) and compound VWM measures (reading, memory recall, mental image manipulation) according to a proposed hierarchically organization of measures.

#### Structural equation modeling

SEM was developed to estimate the direct effect of an independent variable on a dependent variable, in presence of several intra-correlated variables (Wright, 1921). This type of analysis is conceptually analogous to multiple regression models. However, in terms of applications, SEM distinguishes itself notably from these in that the coefficients represent the causal assumptions tested in the model, whereas this is not the case with regression analyses (Myth 2 in Bollen and Pearl, 2013). Another favorable property of SEM is that the method is largely invariant to multicollinearity issues—which multiple regression models are very sensitive to (when independent variables are intra-correlated, individual contributions cannot be distinguished properly). SEM also allows for combined factor-analyses to extract co-varying sources of information as latent variables, which we utilize in the current study to derive common factors for processing speed and VSTM capacity. As in the current study, SEM models are typically specified graphically as hypothesized structures in data (a priori models) that are translated by the SEM software (e.g., LISREL, AMOS), into a hypothetical structure (typically) in the co-variance matrix. The proposed SEM model is then tested against the actual structure in data via optimization (e.g., minimization) of a likelihood function to generate test statistics (typically maximum likelihood estimators) for statistical evaluation.

One of the strongest and most controversial claims surrounding SEM is whether or not causality can be inferred from SEM models. In the current study, we adapt the notion that what SEM does, is to provide quantitative causal conclusions and statistical fit measures based on the qualitative causal assumptions (a priori models), and the empirical measures that are fed into SEM. Further, significant model fit statistics (see the Discussions for an elaboration of these) do not prove the causal assumptions, but makes them tentatively more plausible (Myth 1 in Bollen and Pearl, 2013). In summary, SEM is useful to test relationships between multiple variables and is especially beneficial in large sample studies with several intra-correlated variables, such as in cognitive aging studies here, and in general.

While no previous studies (to our knowledge) have used SEM for the current purpose, related studies have systematically examined the influence of age on visual processing capacity in general. In Verhaeghen and Salthouse (1997) SEM models based on meta analyses of 91 studies showed that while several cognitive measures shared age related variance, the strongest effect of age was found on processing speed. In addition, this effect accounted for a large part of aging effects on more compound/higher level measures. These findings were recently corroborated in a neuroimaging study using SEM to structure the neural implications of aging effects on processing speed and working memory. Although white matter abnormality was only associated with decline in working memory, decline in processing speed was suggested to significantly impact other cognitive abilities (Charlton et al., 2008, however, see Penke and Deary, 2010 for a critique of their methodological approach).

#### A theory of visual attention

To model an assumed hierarchical organization of measures, proper estimates of the fundamental visuospatial functions are required. To this end, we use a whole-report letter paradigm to acquire data which were subsequently modeled according to A Theory of Visual Attention (Bundesen, 1990) for accurate estimates of visual processing/encoding speed (*C*), the capacity of visual short-term memory (*K*) and the visual perceptual threshold (*t0*). TVA is a formal mathematical theory of the fundamental mechanisms of the visual attention system, and provides a computational framework (Kyllingsbæk, 2006; Dyrholm et al., 2011) implemented as a limited capacity parallel race model based on principles of biased competition (Desimone and Duncan, 1995). According to TVA, visual representations race in parallel for encoding into VSTM and both the capacity of VSTM is limited (*K* letters) and the rate with which elements race (*C* letters per second). But the race is biased according to properties of pertinence and relevance to the task, and the probability of winning the race directly depend on these features (e.g., red letter will have a higher probability of encoding compared to a simultaneous presented blue letter if the task is to report red letters), which is formulated in the TVA equations.

TVA modeling has been used in a number of studies, which have justified its empirical relevance. Critical to the purpose of this study, previous findings have established that TVA estimates are sensitive to age, and the encoding/decoding speed to VSTM (processing speed *C*) and VSTM capacity (*K*) decline as we age (Habekost et al., 2012; McAvinue et al., 2012). Similarly, previous studies have shown that TVA estimates are (1) strongly related to commonly accepted neuropsychological measures of matched functions (Finke et al., 2005), and (2) largely unrelated to measures in the Attention Network Task (Posner and Petersen, 1990; Fan et al., 2002), which would be expected (Habekost et al., 2013).

Based on these properties, we employ TVA estimates in the current study and define processing speed by the TVA estimate *C*, and VSTM capacity by *K*, and make the causal assumptions that the TVA estimates constitute the most fundamental measures in the SEM models.

## Methods

### Participants

A total of 112 healthy adults aged 60–75 (*M* = 67.8, *SD* = 4.0) were included in the test. The gender average for age for males was (*N* = 34, *M* = 69.0, *SD* = 3.9) and for females (*N* = 78, *M* = 67.3, *SD* = 3.9), with different educational backgrounds and employment status. The subjects were recruited through the Facebook page of the local branch of The DaneAge Association (“Ældresagen”), an interest group for senior citizens in Denmark, and through advertisements in local newspapers and TV shows. The participants received a courtesy gift of chocolate and wine as thanks for participation. After signing a form of consent, potential participants were directed to a website where they were informed about the inclusion criteria. They had to be healthy with no history of brain injury, dementia and diabetes. They would also be excluded if they currently were under medical treatment for psychiatric disorder or suffered from color blindness. Eyesight (self-reported) had to be normal or corrected to normal.

### Ethical considerations

All participants received oral and written information about the project and their tasks prior to the initiation of the trials. They all signed a written consent form and were instructed that they could leave the project at any time without any explanation.

The study was approved by the regional ethical committee (#40118).

### Cognitive tests

Tests were included in the study based on their sensitivity to measures of visual working memory in general, and age related differences in particular. Furthermore, the test battery was designed with an abstract hierarchal structure of assessment complexity in mind, such that both compound and fundamental measures of visual working memory were included. Top level measures was included to mimic day-to-day activities and serve as test of generalization, while lower- and intermediate-level tests were included to assess visuospatial attention and memory from different angles. As an example, a speed-of-reading task was included as a top-level assessment, while a delayed working memory task was included at the intermediate level and a TVA estimate of processing speed at the lower level. Combined, performance in the reading task are likely to depend on individual performance in the intermediate level task (working memory), which in turn is likely to depend on visual processing speed (TVA estimate *C*) at the lower level (Brown et al., 2012; Franceschini et al., 2012).

Table 1 lists each of the included tests in the test battery with information about the measures they produce as well as the cognitive function being measured and any prior knowledge of sensitivity to age related changes including study references. The Memo task is an exception as it was developed specifically for the current study to provide a generalized measure of a day-to-day memory task. Table 1 also include information about abstraction level of assessment according to the mentioned hierarchy in the tests. In addition to the cognitive tests, a dementia-screening test, the Minimal Mental State Examination (MMSE; Folstein et al., 1975), and a self-developed baseline motor response control test to calculate Fitts parameters (Fitts, 1954) were performed as part of the inclusion criteria to the study.

**Table 1:** **Overview of the testbattery**.

**Test**	**Measures**	**Cognitive function measured**	**Sensitive to age differences**	**Cognitive level**
Delayed Working Memory (interruption condition)	Accuracy of report (percentage)	Processing speed, attentional control	Yes (Clapp et al., 2010)	Intermediate
	Response time (ms)			
Four Mountains (memory condition)	Accuracy of report (percentage)	Attentional control, processing speed, short-term capacity	No but sensitive to cognitive decline in AD (Bird et al., 2009)	Top, intermediate
	Response time (s)			
Corsiblock (forward condition)	Corsispan	Attentional control, processing speed, short-term capacity	Yes for age > 60 (Orsini et al., 1986)	Intermediate
Memo	Completion time	Attentional control, processing speed, short-term capacity	Novel test, no prior existing evidence to our knowledge	Top
	Number of misses			
Reading	Reading time (s)	Attentional control, processing speed, VSTM capacity	Yes (Connelly et al., 1991)	Top
	Accuracy of question report (percentage; only used as control)			
Whole report with TVA modeling	Visual processing speed (C)	Processing speed, short-term capacity	Yes (McAvinue et al., 2012)	Low
	VSTM capacity (K)			
	Visual perceptual threshold (t0)			

All cognitive tests were computerized for ease of application, which also provided bias-free scoring of assessment data. The tests are made freely available under the GNU General Public License and can be acquired from the corresponding author.

### Individual tests

#### Delayed working memory

The test was adapted from Clapp et al. (2010) and the stimuli were provided as courtesy of the main author. It was originally used in different versions to assess sensitivity to manipulated distractor interference in aging. In the current study, we employed the interruption condition from the original test in which a distracting stimulus needs to be attended to while remembering a cue stimulus during the delay phase. The test consisted of 66 trials. In 10% of the trials an interrupting stimulus was included, which required an additional (motor) response. These trials were excluded to avoid response biases on the primary task. In addition, the initial 5 trials were practice trials and were also excluded. A total of 55 trials were included for further analysis.

#### Four mountains task

The test was adapted from Hartley et al. (2007) and computerized with some modifications. In the test participants were required to encode a detailed target stimulus with long exposure time, and immediately after identify the target in a set of 4 test stimuli. However, the target appeared in the test set under manipulated viewing conditions compared to the encoding phase thus imposing strong demands on working memory functions. A written message on the monitor informed participants that a self-paced “spacebar press” initiated a trial. A landscape cue was presented for 8 s immediately followed by a 4-sample probe display where the target would always appear but with viewing conditions manipulated. Selection of target landscape was done using the mouse. The test consisted of 32 trials. The first 5 trials were practice trails were feedback was provided. A total of 27 trials were included for further analysis.

#### Corsispan task

The test was implemented as a forward Corsispan test (Corsi, 1972) with some modifications such as a random tile layout across trials. In the test, participants were required to remember sequences of spatial positions that increased in number of elements across the trials. A written message on the monitor informed participants that a self-paced “spacebar” press initiated a trial. On each trial, 10 purple tiles were randomly distributed across the monitor with the constraints that no linear (neither vertical, diagonal nor vertical) alignments of tiles were formed. During the memory period, individual tiles would lit up (turn yellow) for 1 s with a 1 s inter-tile delay to form a sequences. During the memory period, the mouse cursor was removed and its reintroduction indicated the beginning of the response period. Tiles were selected using the mouse, and a correct response required identification of all previously displayed tiles in the correct sequence. Sequence lengths would span linearly from 2 to 9 with each sequence length being repeated twice. The task terminated when two incorrect reports had been made across all previous trials. Following an incorrect tile selection, the correct sequence of tiles was displayed to participants as feedback. The Corsispan score was the longest sequence that could be correctly reported.

#### Memo task

The test was a novel memorizing procedure where participants had to locate identical pairs of tiles in a static square shaped grid. A 6 × 6 grid of square tiles all with a “?” icon, indicated the beginning of the test. The task was to identify the 18 identical pairs of images of toys hidden behind the 36 tiles. Each tiles was “turned” using the mouse, and upon turning the third tile in sequence, the previous two tiles were turned over again and their identity evaluated for a match. If the two tiles were identical the “?” was replaced with a “√” to indicate a correct pair. The time spend solving the task as well as the number of “misses” were logged at the end and displayed as feedback to the participants.

#### Reading task

The test was adapted from the standard 9 grade Danish reading tests. The texts were extracts from the book “Dyret i dit spejl” by Bent Jørgensen, courtesy of the publisher Gyldendals Forlag. In the reading task, participants read approximately one page of highly factual text, and subsequently answered four questions regarding the text to assert comprehension. A written message on the monitor informed participants that a self-paced “spacebar” press would initiate the task. The completion of reading was recorded by a specific button press, which directed the participant to the 4-question, multiple-choice list. Answer selection was made using the mouse, and upon completion of all four answers participants indicate completion of the task by pressing a “finish” button on the monitor.

#### Whole report task

The test was adapted from (Kyllingsbæk, 2006) specifically to adhere to data format requirements for the TVA modeling procedure. Participants were required to identify—as many as possible—six briefly displayed letter targets arranged on an imaginary circle with an un-speeded response—please see (Wilms and Nielsen, 2014) for a thorough description and illustration of the test. The Whole Report data was used together with the TVA framework to estimate processing speed (*C*), short-term capacity (*K*) and the perceptual threshold (*t*_0_).

#### Fitts screening task (not included in analyses)

The test measured visuomotor performance and required participants to click as fast as possible on one of four cued squares on the screen as fast as possible. Halfway through the test, the size of the squares was reduced to measure the difference in performance (Fitt's value).

#### General protocol

Upon arrival, participants were met and introduced to the place by the test coordinator. Following a brief introduction, participants commenced the first of three test sessions. In the two first test sessions all the cognitive tests were completed, and each of these test session lasted approximately 1 h and 15 min including a 5 min break. In the last test session, participants completed the MMSE screening, which lasted approximately 30 min. Each test was initiated with a tutorial of the test to ensure that all subjects were well-informed. All tests but the MMSE were run on Windows PCs at an approximate viewing distance of 65 cm.

In the first two test sessions, participants were tested together in groups of up to seven people in the same experimental room, and in the last session (the MMSE) participants were tested individually in separate rooms with a test assistant. The three test sessions ran in parallel in separate rooms. Between test sessions, participants were guided to the waiting room where refreshments were made available (coffee, tea, fruit, snacks).

Tests were performed under the supervision of psychology students, who were all thoroughly trained in the test procedures and an experienced MMSE practitioner trained the assistants conducting the MMSE screening (see Acknowledgement Section). All assistants were explicitly informed to make the participants feel comfortable, and talk to them about topics that would come up, except specifics regarding the tests, and analyses. In addition the corresponding author supervised the progression of the sessions and procedures, and were called upon when needed.

### Demographic data

In addition to age, a number of demographic and life style information were gathered using questionnaires. Although some of the information divulged was previously shown to influence the TVA estimates (Wilms and Nielsen, 2014), we have chosen not to include them here since the current purpose is to derive information about the structure in data based on SEM models and the regression of age on specific and general levels of cognition.

### Modeling

#### Structural equation modeling

SEM was used as the primary statistical method to test the causal assumptions made about the structural relations of the measures. The SEM models were specified as graphical models that were translated into testable structures in the covariance matrices of the measures. The basic components of SEM models are the variables and the logical links connecting them. Variables can either be observed/measured variables or unobserved/latent variables (MacCallum and Austin, 2000). Links can either be unidirectional to imply an assumed causal relation (regression), or bidirectional to imply simple correlation (covariance). In addition, each measured- and latent variable may have an associated error/residual variable to account for the (co) variance that cannot be explained by the SEM.

#### Types of SEM and model specification

We use two types of SEM models. Model 1 is a path analysis model where only measured variables are included, whereas Model 2 is a factor analysis model in which latent variables are derived as common factors between the measured variables (MacCallum and Austin, 2000). Models specification of both SEM models presented in the current study follows the *model generation* approach, in which an initial a priori model usually is adapted to the measures in the dataset (Jöreskog and Sörbom, 1996). The consequences of model adaptation to data are discussed in the Discussions Section.

#### SEM statistics

Standardized regression weights β indicate the strength of the (linear) relation (uni-directional links) and imply the direct relation between changes in the connected variables. For instance, A→B, *ß* = −0.3 means that a change in A causes a change in B in the opposite direction, with a magnitude equal to *ß*. Thus, if A increases 1 then B will decrease 0.3. The strength of correlations between variables is estimated by the corresponding covariance between measures. Thus, A↔B = −0.3 means that the co-variation between A and B is 0.3

Both of these statistics are shown in the graphical SEM models next to the links to which they apply.

Regression weights (β) are statistically evaluated by simple *t*-tests based on the critical ratio (CR), which is obtained by dividing a *β*-value with its associated standard error (SE). If the distributional assumptions of normality are met, CR has a standard normal distribution with the null hypothesis that the estimate has a population value of 0. Squared multiple correlations (SMC; *R*^2^) statistics describe the proportion of variance described in a variable by the correspondingly connected variables. Thus, SMC = 0.3 means that 30% of the variance was explained, which also can be used to interpret the residual variance that could not be accounted for by the model.

#### SEM model fit indexes

Several fit indexes are available to assert SEM model fit (Bollen and Long, 1993). Model fit indexes are distinguished on whether they are absolute or relative according to an often-used taxonomy (McDonald and Ho, 2002). Absolute fit indexes compare the proposed structure (the SEM model) to the actual one in data (here the covariance matrix) by minimization of a likelihood function to produce maximum likelihood (ML) estimators (Browne, 1984). Relative fit indexes are based on comparison of the ML estimators of the fitted model and a null model with uncorrelated variables (McDonald and Ho, 2002).

In the current study, we report two popular absolute fit indexes to evaluate how well the SEM models fit the data. The chi-square test of the ML estimators assert the probability that a more complex model fit the data better (McDonald and Ho, 2002). It is based on central distributional assumptions, and the null hypothesis stating that there is no difference between the proposed structure in the covariance matrix and the actual one. Thus, non-significant chi-square statistics suggest that the data fits well to the proposed SEM model and favors acceptance of the model. We also report the Root Mean Square Error of Approximation (RMSEA; Steiger and Lind, 1980; Browne and Cudeck, 1992), which is currently the most popular model fit index (Kenny et al., 2014). RMSEA is based on the non-centrality parameter (here the chi-square function subtracted the degrees of freedom) and that the null is false. Thus, the significance level of a statistical RMSEA evaluation implies how well the model fits the data. While criteria in the range [0.01–0.08] have been proposed to indicate excellent to mediocre fits (MacCallum et al., 1996) recent report criticize the use of cut-off criteria on the basis of lack of empirical support of these (Chen et al., 2008). However, general praise for RMSEA is mediated by the availability of confidence intervals, which relaxes assumptions on cut-off criteria (Hu and Bentler, 1998; MacCallum and Austin, 2000).

#### TVA

The whole-report data were fitted with the TVA framework (Bundesen, 1990) using MATLAB and the LibTVA toolbox (Dyrholm et al., 2011) to extract the *t*_0_, *C* and *K* parameters. The LibTVA toolbox and user guide can freely be downloaded from http://zappa.psy.ku.dk/libtva.

### Data analysis

Raw response data were pre-processed in Python according to the principles described below. Statistical analyses were performed using IBM SPSS V.20 and IBM SPSS Amos V22.

### Outlier detection

We introduce a formal automated pipeline for detecting outliers to eliminate the manual tedious work of eyeballing data, and reduce the potential for biases. Outliers are detected at the trial level and at the participant level. Outlier detection on both trial and participant level is based on the Median Absolute Deviation method (MAD; see Leys et al., 2013 for a recent, relevant application). The MAD is an average measure of the variation of data points relative to the median. Specific data points are detected as outliers based on a ±2.5 MAD filter. The advantage of using the median as that the central point of evaluation is less influenced by outliers than when the mean is used, which is true both when computing the MAD and when detecting outliers based on the MAD. The outlier pipeline also includes a modified square root transformation (Cousineau and Chartier, 2010) to impose normality on response time (RT) measures that are inherently skewed. This procedure is applied at trial level only.

The outlier pipeline replaces formal guessing-rate criteria that can otherwise be used to control for random responses. For instance, the DWM task has a guessing rate of 0.25 and participants that fall near this threshold should be excluded on the account of random responses. In the current dataset, the outlier pipeline detected all of the cases where responses were near the guessing threshold by means of the MAD filter mentioned above. This approach thus resolved the issue of formally evaluating when a data point is statistically significant from guessing.

Outlier detection on trial level RTs were performed for both correct and incorrect response trials, and the subject level RT scores were computed as the average of all the trials surviving this procedure. This approach differs from the more traditional one used in some studies where the average RT scores are computed from correct response trials only (e.g., Clapp et al., 2010). The rationale behind using correct response trials only is that incorrect response trials may confound the average RT score by means of inattentiveness to the task. While this argument is valid when response accuracies are near ceiling, it is less so when task difficulty directly influences the response accuracy, such as in the Four Mountains- and DWM tasks used here.

Outlier detection at subject level was performed on the individual each test measures, and outlier identification of one measure within a test automatically excluded all measures within that test (pair-wise exclusion). This conditional criterion prevents confounds from random and flawed response. For instance, if participants misunderstood the DWM task instructions and due to this miscomprehension entered incorrect responses, it would yield a valid but random RT measure, but an invalid accuracy measure (where valid refers to whether they were detected by the outlier pipeline). In such cases both DWM measures would be excluded.

#### Missing values exclusion

Missing values were handled in SPSS by pairwise deletion, which contrary to list-wise deletion preserves the valid variables for a participant. AMOS on the other hand, is able to compute ML estimators even when values are missing based on principles by Anderson (1957).

## Results

First, normative data is presented to provide an overview of the distributional properties of the cognitive measures in the dataset. Secondly, a set of bivariate correlations is presented to provide an overview of the correlation within the dataset, and to provide a means to test alternative SEM models for the dataset according to the principles described by Holm (1979; see Penke and Deary, 2010 for a more recent application). Finally, the SEM models are presented, which constitute the main results of the study from which we draw inferences and make conclusions.

### Test scores and correlational statistics

Table 2 presents the descriptive test score statistics, and in Table 3 the correlational statistics (Pearson's *r*) are presented. The purpose of the data tables is to give an overview of the dataset, how it is correlated, and how baseline visuomotor differences—as measured by response time on the Fitts test—influence the measures.

**Table 2:** **Descriptive statistics for the test scores**.

	***N***	**Mean**	***SD***
DWM_Acc	104	0.69	0.14
DWM_RT	104	1468	354
FM_Acc	107	0.66	0.14
FM_Time	107	11.5	3.5
Corsi_Span	112	5	1
Memo_Time	99	222	58
Read_Time	103	249	64
TVA_t0	93	21.9	14.5
TVA_C	93	43.21	16.54
TVA_K	93	3.58	0.67
Fitts_RT	110	503	8

**Table 3:** **Correlation analysis (Pearson's *r*) of the dataset**.

		**Age**	**Corsi_Span**	**DWM_Acc**	**DWM_RT**	**Fitts_RT**	**FM_Acc**	**FM_ Time**	**Memo_Time**	**Read_Time**	**TVA_t0**	**TVA_C**
Corsi_Span	*r*	0.140	1									
	*N*	112	112									
DWM_Acc	*r*	0.021	0.054	1								
	*N*	104	104	104								
DWM_RT	*r*	0.266	−0.022	−0.023	1							
	*N*	104	104	104	104							
Fitts_RT	*r*	0.024	0.191	−0.067	.035	1						
	*N*	110	110	103	103	110						
FM_Acc	*r*	0.133	0.308^**^	0.308^*^	−0.033	0.130	1					
	*N*	107	107	101	101	107	107					
FM_Time	*r*	0.167	0.080	−0.009	0.030	0.062	0.088	1				
	*N*	107	107	101	101	107	107	107				
Memo_Time	*r*	0.205	0.329^**^	0.009	0.3^*^	−0.098	0.271	0.025	1			
	*N*	99	99	92	92	97	95	95	99			
Read_Time	*r*	0.274	−0.042	−0.022	0.304^*^	0.060	0.150	0.222	0.178	1		
	*N*	103	103	97	97	103	102	102	92	103		
TVA_t0	*r*	0.051	0.010	−0.019	0.085	0.037	0.002	0.011	0.141	0.033	1	
	*N*	93	93	89	89	91	90	90	82	87	93	
TVA_C	*r*	0.166	0.238	0.092	−0.199	0.172	0.150	0.034	−0.298	−0.115	0.132	1
	*N*	93	93	89	89	91	90	90	82	87	93	93
TVA_K	*r*	0.054	0.250	−0.026	−0.124	0.115	0.114	0.092	−0.249	−0.003	−0.230	0.384
	*N*	93	93	89	89	91	90	90	82	87	93	93

* *Correlation is significant at the 0.05 level (2-tailed) Bonferroni corrected for multiple comparisons*.

** *Correlation is significant at the 0.01 level (2-tailed) Bonferroni corrected for multiple comparisons*.

The missing values in Table 2 (the deviation from *N* = 112) reflect the filtering by the above-mentioned outlier detection pipeline. Outlier detection excluded an average of 6% of trials (*M* = 3.6; *SD* = 2.9) for the DWM_RT measure, and 3% of the trials (*M* = 0.86; *SD* = 1.2) for the FM_Time measure. Outlier detection on subject level excluded TVA measures for 14 participants, Memo measures for 13 participants, Reading measures for 6 participants, and Four Mountain's measures for 3 participants, while no outliers were detected in the Corsispan and DWM tasks. TVA measures were also excluded if the relative weight ratio between the left and the right side of the monitor fell outside the range 0.3–0.7 implying a strong bias to either side. This laterality bias could limit the estimate of *K* if participants had focused only on a small subset of the letters (e.g., Duncan et al., 1999). A total of 6 participants (part of the already mentioned 14 participants) were excluded on this criterion, and the average TVA *K* estimate of these *M* = 2.46 (*SD* = 0.04), compared to the average of all the included participants *M* = 3.58 (*SD* = 0.67) supports the argument of limited information to properly estimate *K*.

No effect of age was evident on any of the measures at the individual test level. That age did not influence the TVA estimates for processing speed (*C*), and for VSTM capacity (*K*) is not consistent with other similar studies (e.g., McAvinue et al., 2012), but consistent with what we previously found when adjusting for demographic and lifestyle factors (Wilms and Nielsen, 2014).

In Table 3, Fitts_RT correlations assert the influence of baseline visuomotor differences on the cognitive tasks. Inspection of the table indicates that the cognitive measures were independent of the Fitts scores. Thus, the Fitts_RT measure is omitted from further analyses.

The accuracy of response to questions in the reading test was excluded due to an extreme asymmetrical distribution (kurtosis = 3.45) and a sparse continuity (100% divided in 4 levels), which prevents efficient transformation of the measures (Cousineau and Chartier, 2010). The number of misses in the Memo task was excluded due to its strong dependency on the completion time of the Memo task, which is the primary measure of the task.

### Structural equation models

In the SEM models, standardized regression weights and covariance estimates are presented on the corresponding links, and the squared multiple correlation (*R*^2^) estimates on the variables. Unless otherwise specified, the only constraints imposed on the models are those of the residual regression weights (initially set to 1), which is more a convention than a constraint. Model fit statistics for the chi-square and the RMSEA indexes are presented in the models in a standard formats (note that LOW and UPPER in the RMSEA statistics correspond to the lower and upper 90% confidence intervals of the RMSEA index). Model-fit statistics for both SEM models encourage acceptance of fits of the models to the dataset. In addition, unless otherwise noted all regression statistics are significant at least at the 0.05 level.

#### Model 1: hierarchical and functional dependency structures

**Figure 1:**
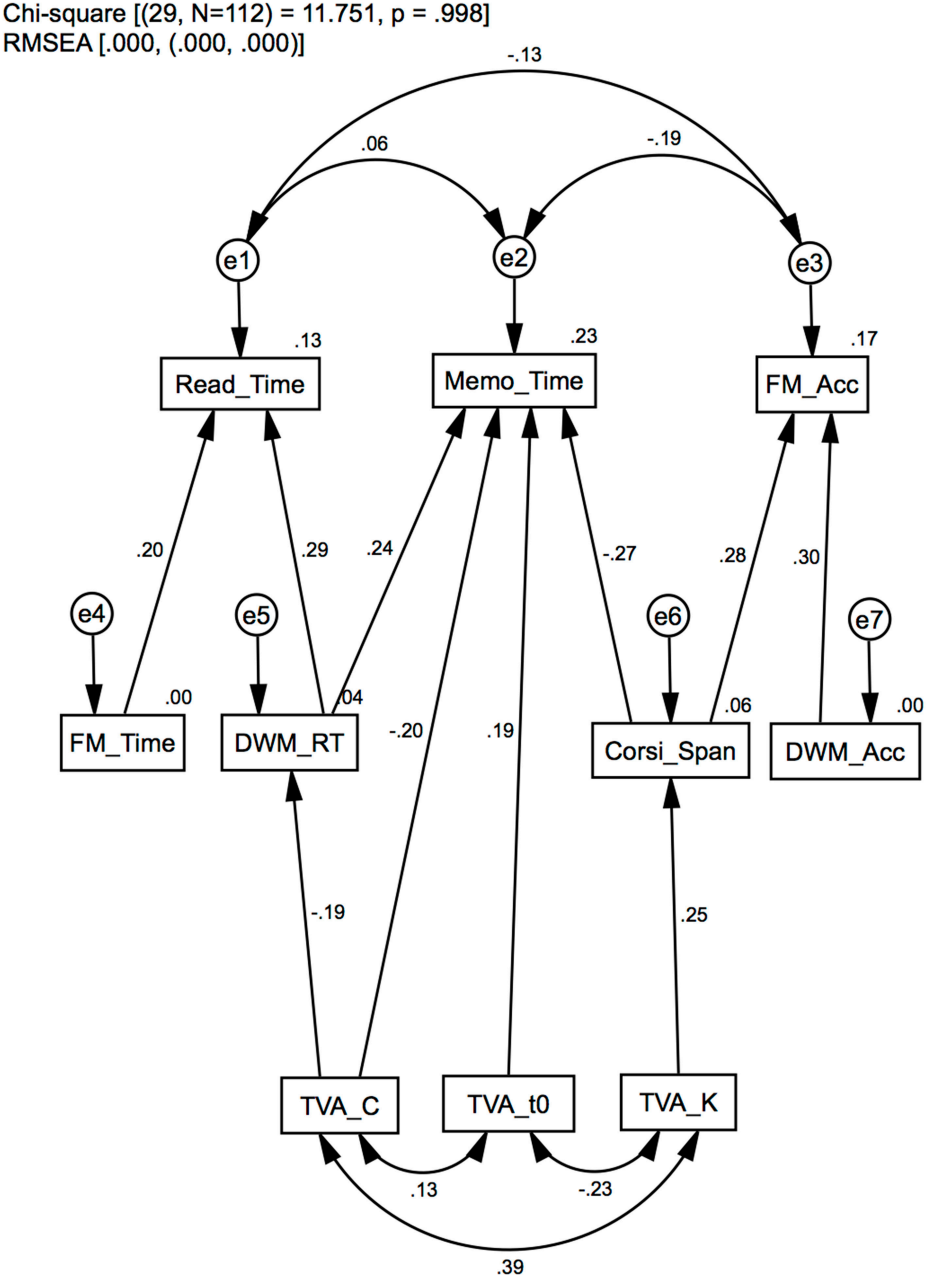
**Model 1: Standardized path analysis**. Hierarchical dependencies in layers according to functional complexity, and in distinct structures according to functional specificity: processing speed (left), VSTM capacity (right) or a combination (middle). Standardized regression weights and covariance estimates are presented on the corresponding links, and squared multiple correlation (*R*^2^) estimates on the variables.

Model 1 is illustrated in Figure 1. The purpose of this model is to test an a priori model of how the measures in the dataset are structured in terms of level of assessment complexity and dependency on the two cognitive functions in question. In Model 1, branches originate from the fundamental estimates of TVA through intermediate levels, and terminates at the three top-level VWM measures: completion time in the Reading task (Read_Time), completion time on the Memo task (Memo_Time), and response accuracy in the Four Mountains task (FM_Acc). The leftmost structure in the model (with its apex at Read_Time) comprises measures most strongly related to processing speed [TVA_C, DWM_RT, FM_Time, Read_Time], the rightmost one to measures of VSTM capacity [TVA_K, Corsi_Span, DWM_Acc, FM_Acc], while the middle structure indicate measures that depend more equally on both cognitive functions [TVA_C, DWM_RT, TVA_K, TVA_t0, Corsi_Span, Memo_Time]. All but the TVA_C→DWM_RT (*p* = 0.07) regression weights were significant. The average significance level for all regression coefficients was *M* = 0.02 (*SD* = 0.02). Covariance statistics suggest a strong correlation between TVA_K↔TVA_C (*p* < 0.01) and a modest correlation between TVA_K↔TVA_t0 (*p* < 0.05), which we have reported in a previous article (Wilms and Nielsen, 2014), and which are common to TVA estimates in general (e.g., McAvinue et al., 2012). The residual variances for the high level measures were uncorrelated, although e2↔e3 was marginally significant (*p* = 0.08). In summary, Model 1 shows that measures can be structured according to their level of assessment complexity, which is consistent with previous findings (Brown et al., 2012; Franceschini et al., 2012). Furthermore, the functional organization of measures indicates two major structures of processing speed and VSTM capacity.

#### Model 2: aging effects on processing speed and VSTM capacity

**Figure 2:**
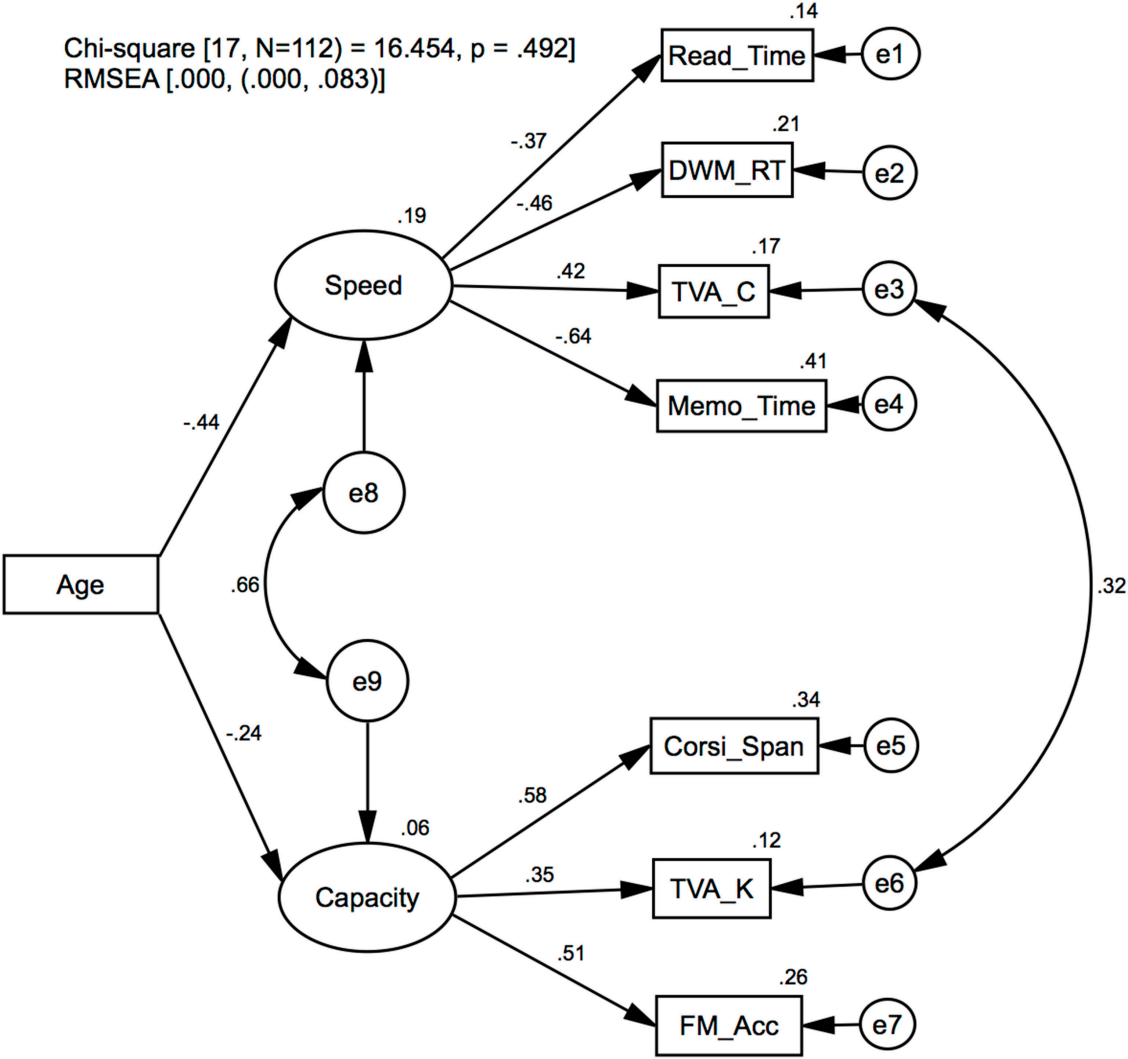
**Model 2: Confirmatory factor analysis**. Latent variables for processing speed (Speed) and VSTM capacity (Capacity), and the regression of age on these. Standardized regression weights and covariance estimates are presented on the corresponding links, and the squared multiple correlation (*R*^2^) estimates on the variables.

Model 2 is illustrated in Figure 2. The purpose of the model is to test whether more general estimates of processing speed and VSTM capacity can be derived from multiple measures and how aging affects these. Model specification was informed by the functional structures in Model 1 to extract common factors based on the subset of data that were most strongly related to either processing speed [Speed→(Read_Time, DWM_RT, TVA_C, Memo_Time)] or VSTM capacity [Capacity→(Corsi_Span, TVA_K, FM_Acc)]. The factors were modeled as exogenous latent variables that on the one hand influence the dependent measured variables and on the other hand are influenced by Age and their respective residual variables (e8, e9). To enforce the integrity of the factors in representing their corresponding cognitive functions, the TVA measures *C* and *K* were assumed to define each function by constraining the model with the factor loadings TVA_C→Speed and TVA_K→Capacity, explicitly set to 1. To model the commonly found correlation between TVA estimates, *C* and *K* (see Model 1 and description), the covariance was estimated between the residuals of those variables (e3↔e6). The average significance level for all factor loadings originating from Speed and Capacity was *M* = 0.02 (*SD* = 0.01). Covariance estimates for the residuals pertaining to Speed and Capacity suggests that independent factor were extracted to represent these cognitive functions (*p* = 0.12). Furthermore, the TVA_K and TVA_C variables were correlated as indicated by significant covariance estimates for their respective residuals (e3↔e6, *p* = 0.01).

Age significantly affected Speed (*p* = 0.01), while Capacity was not significantly affected (*p* = 0.14). These findings are consistent with some findings suggesting that processing speed and not capacity is influenced by age (e.g., Brown et al., 2012) but inconsistent with other findings suggesting that both are affected by age (e.g., McAvinue et al., 2012).

In summary, Model 2 shows that common factors can be derived to estimate more general representations of processing speed and VSTM capacity, and while age did not influence the TVA estimates for processing speed (see Table 3) as it has been reported elsewhere, we found a more general effect of age on processing speed components (Salthouse, 1996, 2000).

## Discussions

### Summary

In the current study, we examined the influence of age on behavioral measures of visuospatial attention and memory in a sample of 112 healthy adults aged 60–75. Model 1 provides evidence for a hierarchical dependency structure between measures according to functional complexity, such that higher-level visual working memory (VWM) performance could be predicted by several performance measures of lower-level visuospatial functions. Furthermore, measures were grouped in distinct structures according to functional specificity. Measures relating to processing speed were grouped in a left side structure and measures relating to VSTM capacity in a right side one, while a central structure was indicative of measures depending more equally on both functions. In Model 2, the effect of age was assessed on common factors for processing speed (Speed) and VSTM capacity (Capacity) that were derived from several distinct measures based on Model 1. In summary, our results suggest that for this sample, processing speed more than VSTM capacity is affected by age.

### On the influence of age

The main findings on the effects of aging are that processing speed more than VSTM capacity is affected by age. Moreover, in previous regression analyses on the same dataset we were not able to detect aging effects on any of the TVA estimates (Wilms and Nielsen, 2014) (see also Table 3) as it has previously reported (Habekost et al., 2012; McAvinue et al., 2012; Espeseth et al., 2014). However, here we were able to detect significant age effect on the latent construct for processing speed (Speed; Model 2) when the TVA estimate for processing speed (*C*) was assumed to depend on Speed. In effect, Model 2 showed that although the TVA *C* parameter was not influenced by age at the individual test level, the processes represented by TVA *C* might in fact be. In conclusion, the SEM approach presented here provides a sensitive method to examining cognitive aging effects, even within a relatively narrow age-range.

The finding of selective effects of age on processing speed, compared to VSTM capacity, is consistent with some studies (Salthouse, 2000; Hedden and Gabrieli, 2004; Brown et al., 2012) and inconsistent with others reporting aging effects on both TVA measures for processing speed (C) and VSTM capacity (K; Habekost et al., 2012; McAvinue et al., 2012; Espeseth et al., 2014). There are several plausible explanations to this inconsistency. In the previous TVA studies participants belonged to older age groups and broader age ranges than in our study, which may have led to a larger age related variance in these studies compared to ours. Similarly, differences in demographic- and lifestyle variables between the samples may alter the onset age of decline in addition to cause different trajectories for the age effects. A general review by Hedden and Gabrieli (2004) points to the fact that the onset age of decline is highly individual and dependent on many factors, such as education level and social engagement level. Even cognitive engagement has been found to have a substantial influence (Wilson et al., 2012). However, one of the main findings in relation to cognitive memory decline are remarkable sparing of semantic and short-term memory with age, contrasted by a decline in autobiographical, emotional and implicit memory (Hedden and Gabrieli, 2004). This would support the findings in this study as well as in our previous study (Wilms and Nielsen, 2014) that the capacity estimates of VSTM is more robust to age-related changes in healthy adults, at least within this narrow age range. Processing speed, on the other hand, has for a long time been considered to be declining at a general level from 40 years and onwards (Salthouse, 2000).

### On the correlation between the TVA C and K estimates

A typical finding in the TVA literature is that the estimates for processing speed, *C*, and VSTM capacity, *K* are correlated (e.g., Finke et al., 2005; Habekost et al., 2013). In Wilms and Nielsen (2014) we also reported this finding, however, here we were able to model the correlation as the covariance between the residuals for TVA estimates (Model 2) and to extract independent common factors for processing speed and VSTM capacity on the same dataset. The correlation between TVA estimates does not pose a problem for the theoretical validity of TVA, since there are several plausible explanations as to why individuals with a relative high VSTM capacity tend to have a comparable high processing speed vice versa (e.g., common underlying resources). Nevertheless, we find it encouraging being able model the correlation at measurement level and examining the constructs independently—whatever the source of correlation may be.

### On the interpretation and evaluation of the SEM models

A number of limitations arise when making inferences based on individual SEM models/datasets, which relate to generalizability of findings and thus the conclusions that can be drawn from them. Interpretations are frequently criticized, and cautions raised in the SEM literature that one can only strive to propose an adequate model, which fits reasonably well to data. Even when the model fits the data extremely well, this does not strengthen generalizability but merely indicates that the model is plausible (MacCallum and Austin, 2000). To further strengthen the plausibility of *a* SEM model, it should be critically evaluated against alternative SEM models (model comparison), and preferably on different datasets (Bollen and Pearl, 2013).

Evaluation of SEM models are based on model fit indexes and while a large number of these have been proposed (e.g., Bollen and Long, 1993), there is little consistent advice about which index asserts a given type of model best (MacCallum and Austin, 2000). This lack of standard may have caused a trend toward merely reporting a large number of these indexes “apparently because we don't know how to use any of them” (McDonald and Ho, 2002). A recent discussion on the topic in a supplementary SEM module to the American Psychology Associations' (APA) Journal Article Reporting Standards (JARS; Hoyle and Isherwood, 2013) also reflects this inconsistency. Despite the authority of this institution in conceiving clear recommendations, there are no direct suggestions, and it is merely emphasized that authors should critically review the core literature. In the endeavor of aligning our approach most efficiently, we reviewed the articles discussed there along with the cited literature in those. Ultimately, we based our approach on the highly influential reviews of MacCallum and Austin (2000) and Hu and Bentler (1998), on specific advices to the Psychological literature (McDonald and Ho, 2002), and on those that generally concern best practice in advanced SEM studies (Mueller and Hancock, 2008; Bollen and Pearl, 2013).

The two model fit indexes reported here (see Methods section for description) are sensitive to sample size to an extend that requires substantial consideration when interpreting them (which goes for goodness of fit indexes in general). A relatively large sample size will cause even insignificant discrepancies to be significant for the chi-square test of the ML estimate (Hox and Bechger, 1998). Furthermore, since the RMSEA is a non-centrality estimate based on the chi-square test of the ML estimate, by implication the same argument goes for the RMSEA, although with the opposite effect of sample size. Thus, the two fit indexes may be influenced equally by sample size and degrees of freedom (due to their mathematical equality) but with opposite signs in terms of model rejection (due their opposing statistical characteristics). More importantly, in these boundary conditions, the integrity of these fit indexes are controversial as they convey little useful information. While there is no specific definition on what constitutes *small* sample sizes or degrees of freedom, it is the authors' interpretation based on (MacCallum and Austin, 2000) that fewer than 100 samples might be critical (however see Barrett, 2007 for an even more critical approach suggesting 200 as a limit). This proposal is quantitatively supported by a recent simulation approach by Kenny et al. (2014) suggesting that a sample size of 50 or below is critical especially for small degrees of freedom (approximately < 10), whereas a sample size of 100 in combination with 10 or more degrees of freedom are sufficient. In accordance with the above, we suggest that data conditions were proper in the current study and that the SEM models proposed do fit the data well.

The design of the SEM models presented here can be categorized as belonging to the *model generation* strategy (Jöreskog and Sörbom, 1996) in which an initial a priori model is adapted to the measures in the dataset. According to a thorough review of 500 publications in 16 psychology journals between 1993 and 1997 MacCallum and Austin (2000) found that this strategy was used in approximately 25% of the studies reviewed, and argued that this number is unfortunately high. According to the authors, the problem with model generation strategies lies in its data-driven approach, which, in combination with the mentioned issues of specificity to dataset, further challenges the generalizability of SEM models. The proposed optimal strategy for evaluating SEM models is the *alternative models* strategy (Jöreskog and Sörbom, 1996) where several a priori models are tested against each other and conclusions are based strictly on which model was the best predictor of the dataset, more than which model was the most correct one. To acknowledge this procedure and best practice we relax our assumptions of generalizability accordingly, and have reported the changes made from the original a priori models. Modifications were modest for Model 1 and included only reorganizing of 10–15% of links and introduction of additional covariance links due to lack of initial technical understanding (more than a conceptual reorganization of the model). For Model 2, the modifications required removal of an unobserved variable for attentional control and reorganizing of 20–30% of links over 2–3 iterations since there were not enough power in the data set to derive a robust factor relating to attentional control. Although several of the tests in our study impose strong demands on attentional control (e.g., Corsispan task) we did not intend to assess attentional control explicitly and as such did not include distinct measures of these functions, which is a likely cause as to why no construct could be derived for attentional control (however, see Salthouse et al., 2003 for an alternative explanation).

## Conclusions

We studied aging effects on behavioral measures of visuospatial attention and memory in 112 healthy adults between 60 and 75 years of age. Using Structural Equation Modeling (SEM), we were able to model the hierarchical dependency structures between higher and lower level measures according to functional complexity, and distinct structures that grouped measures according functional specificity (Model 1). Furthermore, based on distinct measures of either function (informed by Model 1) we were able to derive independent latent constructs for processing speed and visual short-term (VSTM) capacity, and examine the effect of age on these (Model 2). The main finding is that processing speed compared to VSTM capacity is most strongly influenced by age in this sample. The current study also demonstrated that the proposed SEM framework is a sensitive approach to detect even subtle cognitive changes within a narrow age range.

### Conflict of interest statement

The authors declare that the research was conducted in the absence of any commercial or financial relationships that could be construed as a potential conflict of interest.

## References

[B1] Anderson N. D. Craik F. I. Naveh-Benjamin M. (1998). The attentional demands of encoding and retrieval in younger and older adults: I. Evidence from divided attention costs. Psychol. Aging 13:405. 10.1037/0882-7974.13.3.405 9793117

[B2] Anderson T. W. (1957). Maximum likelihood estimates for a multivariate normal distribution when some observations are missing. J. Am. Stat. Assoc. 52, 200–203 10.1080/01621459.1957.10501379

[B3] Anguera J. A. Boccanfuso J. Rintoul J. L. Al-Hashimi O. Faraji F. Janowich J.. (2013). Video game training enhances cognitive control in older adults. Nature 501, 97–101. 10.1038/nature12486 24005416 PMC3983066

[B4] Barrett P. (2007). Structural equation modelling: adjudging model fit. Pers. Individ. Dif. 42, 815–824 10.1016/j.paid.2006.09.018

[B5] Berg C. Hertzog C. Hunt E. (1982). Age differences in the speed of mental rotation. Dev. Psychol. 18:95 10.1037/0012-1649.18.1.95

[B6] Bird C. M. Chan D. Hartley T. Pijnenburg Y. A. Rossor M. N. Burgess N. (2009). Topographical short-term memory differentiates Alzheimer's disease from frontotemporal lobar degeneration. Hippocampus 20, 1154–1169. 10.1002/hipo.20715 19852032

[B7] Bollen K. A. Long J. S. (1993). Testing Structural Equation Models. Thousand Oaks, CA: SAGE.

[B8] Bollen K. A. Pearl J. (2013). Eight myths about causality and structural equation models, in Handbooks of Sociology and Social Research, ed Morgan S. L. (Dordrecht: Springer), 301–328.

[B9] Brown L. A. Brockmole J. R. Gow A. J. Deary I. J. (2012). Processing speed and visuospatial executive function predict visual working memory ability in older adults. Exp. Aging Res. 38, 1–19. 10.1080/0361073X.2012.636722 22224947

[B10] Browne M. W. (1984). Asymptotically distribution−free methods for the analysis of covariance structures. Br. J. Math. Stat. Psychol. 37, 62–83. 10.1111/j.2044-8317.1984.tb00789.x 6733054

[B11] Browne M. W. Cudeck R. (1992). Alternative ways of assessing model fit. Sociol. Methods Res. 21, 230–258 10.1177/0049124192021002005

[B12] Bundesen C. (1990). A theory of visual attention. Psychol. Rev. 97, 523–547. 10.1037/0033-295X.97.4.523 2247540

[B13] Charlton R. A. Landau S. Schiavone F. Barrick T. R. Clark C. A. Markus H. S.. (2008). A structural equation modeling investigation of age-related variance in executive function and DTI measured white matter damage. Neurobiol. Aging 29, 1547–1555. 10.1016/j.neurobiolaging.2007.03.017 17451845

[B14] Clapp W. C. Gazzaley A. (2012). Distinct mechanisms for the impact of distraction and interruption on working memory in aging. Neurobiol. Aging 33, 134–148. 10.1016/j.neurobiolaging.2010.01.012 20144492 PMC2891289

[B15] Clapp W. C. Rubens M. T. Gazzaley A. (2010). Mechanisms of working memory disruption by external interference. Cereb. Cortex 20, 859–872. 10.1093/cercor/bhp150 19648173 PMC2837090

[B16] Connelly S. L. Hasher L. Zacks R. T. (1991). Age and reading: the impact of distraction. Psychol. Aging 6:533. 10.1037/0882-7974.6.4.533 1777141

[B17] Corsi P. M. (1972). Human Memory and the Medial Temporal Region of the Brain. Ph.D. thesis, Department of Psychology, McGill University, Montreal.

[B18] Cousineau D. Chartier S. (2010). Outliers detection and treatment: a review. Int. J. Psychol. Res. 3, 58–67.

[B19] Desimone R. Duncan J. (1995). Neural mechanisms of selective visual attention. Annu. Rev. Neurosci. 18, 193–222. 10.1146/annurev.ne.18.030195.001205 7605061

[B20] Dror I. E. Kosslyn S. M. (1994). Mental imagery and aging. Psychol. Aging 9:90. 10.1037/0882-7974.9.1.90 8185873

[B21] Duncan J. Bundesen C. Olson A. Humphreys G. Chavda S. Shibuya H. (1999). Systematic analysis of deficits in visual attention. J. Exp. Psychol. Gen. 128, 450–478. 10.1037/0096-3445.128.4.450 10650583

[B22] Dyrholm M. Kyllingsbæk S. Espeseth T. (2011). Generalizing parametric models by introducing trial-by-trial parameter variability: The case of TVA. J. Math. Psychol. 55, 416–429 10.1016/j.jmp.2011.08.005

[B23] Espeseth T. Vangkilde S. Petersen A. Dyrholm M. Westlye L. (2014). TVA–based assessment of attentional capacities–associations with age and indices of brain white matter microstructure. Front. Psychol. 5:1177. 10.3389/fpsyg.2014.01177 25374549 PMC4204453

[B24] Fan J. McCandliss B. D. Sommer T. Raz A. Posner M. I. (2002). Testing the efficiency and independence of attentional networks. J. Cogn. Neurosci. 14, 340–347. 10.1162/089892902317361886 11970796

[B25] Chen F. Curran P. J. Bollen K. A. Kirby J. Paxton P. (2008). An empirical evaluation of the use of fixed cutoff points in RMSEA test statistic in structural equation models. Sociol. Methods Res. 36, 462–494. 10.1177/0049124108314720 19756246 PMC2743032

[B26] Finke K. Bublak P. Krummenacher J. Kyllingsbæk S. Müller H. J. Schneider W. X. (2005). Usability of a theory of visual attention (TVA) for parameter-based measurement of attention I: evidence from normal subjects. J. Int. Neuropsychol. Soc. 11, 832–842. 10.1017/S1355617705050976 16519262

[B27] Fitts P. M. (1954). The information capacity of the human motor system in controlling the amplitude of movement. J. Exp. Psychol. 47, 381–391. 10.1037/h0055392 13174710

[B28] Folstein M. F. Folstein S. E. McHugh P. R. (1975). Mini-mental state. J. Psychiatr. Res. 12, 189–198 10.1016/0022-3956(75)90026-6 1202204

[B29] Franceschini S. Gori S. Ruffino M. Pedrolli K. Facoetti A. (2012). A causal link between visual spatial attention and reading acquisition. Curr. Biol. 22, 814–819. 10.1016/j.cub.2012.03.013 22483940

[B30] Gazzaley A. Cooney J. W. Rissman J. D'Esposito M. (2005). Top-down suppression deficit underlies working memory impairment in normal aging. Nat. Neurosci. 8, 1298–1300. 10.1038/nn1543 16158065

[B31] Habekost T. Vogel A. Rostrup E. Bundesen C. Kyllingsbæk S. Garde E.. (2012). Visual processing speed in old age. Scand. J. Psychol. 54, 89–94. 10.1111/sjop.12008 23121639

[B32] Habekost T. Petersen A. Vangkilde S. (2013). Testing attention: comparing the ANT with TVA-based assessment. Behav. Res. Methods 46, 81–94. 10.3758/s13428-013-0341-2 23592299

[B33] Hartley J. T. Stojack C. C. Mushaney T. J. Annon T. A. Lee D. W. (1994). Reading speed and prose memory in older and younger adults. Psychol. Aging 9:216. 10.1037/0882-7974.9.2.216 8054169

[B34] Hartley T. Bird C. M. Chan D. Cipolotti L. Husain M. Vargha-Khadem F.. (2007). The hippocampus is required for short-term topographical memory in humans. Hippocampus 17, 34–48. 10.1002/hipo.20240 17143905 PMC2677717

[B35] Hedden T. Gabrieli J. D. E. (2004). Insights into the ageing mind: a view from cognitive neuroscience. Nat. Rev. Neurosci. 5, 87–96. 10.1038/nrn1323 14735112

[B36] Holm S. (1979). A simple sequentially rejective multiple test procedure. Scand. J. Stat. 6, 65–70.

[B37] Hox J. J. Bechger T. M. (1998). An introduction to structural equation modelling. Fam. Sci. Rev. 11, 354–373.

[B38] Hoyle R. H. Isherwood J. C. (2013). Reporting results from structural equation modeling analyses in archives of scientific psychology. Arch. Sci. Psychol. 1, 14–22. 10.1037/arc0000004 23997997 PMC3755633

[B39] Hu L.-T. Bentler P. M. (1998). Fit indices in covariance structure modeling: sensitivity to underparameterized model misspecification. Psychol. Methods 3:424 10.1037/1082-989X.3.4.424

[B40] Jöreskog K. G. Sörbom D. (1996). LISREL 8. Lincolnwood, IL: Scientific Software International.

[B41] Kenny D. A. Kaniskan B. McCoach D. B. (2014). The performance of RMSEA in models with small degrees of freedom. Sociol. Methods Res. [Epub ahead of print]. 10.1177/0049124114543236

[B42] Kyllingsbæk S. (2006). Modeling visual attention. Behav. Res. Methods 38, 123–133. 10.3758/BF03192757 16817521

[B43] Leys C. Ley C. Klein O. Bernard P. Licata L. (2013). Detecting outliers: do not use standard deviation around the mean, use absolute deviation around the median. J. Exp. Soc. Psychol. 49, 764–766 10.1016/j.jesp.2013.03.013

[B44] MacCallum R. C. Austin J. T. (2000). Applications of structural equation modeling in psychological research. Annu. Rev. Psychol. 51, 201–226. 10.1146/annurev.psych.51.1.201 10751970

[B45] MacCallum R. C. Browne M. W. Sugawara H. M. (1996). Power analysis and determination of sample size for covariance structure modeling. Psychol. Methods 1, 130–149. 10.1037/1082-989X.1.2.13019059362

[B46] McAvinue L. P. Habekost T. Johnson K. A. Kyllingsbæk S. Vangkilde S. Bundesen C.. (2012). Sustained attention, attentional selectivity, and attentional capacity across the lifespan. Atten. Percept. Psychophys. 74, 1570–1582. 10.3758/s13414-012-0352-6 22825931

[B47] McDonald R. P. Ho M.-H. R. (2002). Principles and practice in reporting structural equation analyses. Psychol. Methods 7, 64–82. 10.1037/1082-989X.7.1.64 11928891

[B48] Mueller R. O. Hancock G. R. (2008). Best practices in structural equation modeling, in Best Practices in Quantitative Methods, ed Osborne J. W. (Louisville, KY: SAGE Publications, Inc.), 488–508.

[B49] Orsini A. Chiacchio L. Cinque M. Cocchiaro C. Schiappa O. Grossi D. (1986). Effects of age, education and sex on two tests of immediate memory: a study of normal subjects from 20 to 99 years of age. Percept. Mot. Skills 63, 727–732. 10.2466/pms.1986.63.2.727 3808855

[B50] Penke L. Deary I. J. (2010). Some guidelines for structural equation modelling in cognitive neuroscience: the case of Charlton et al.'s study on white matter integrity and cognitive ageing. Neurobiol. Aging 31, 1656–1660. 10.1016/j.neurobiolaging.2009.10.019 20079555

[B51] Posner M. L. Petersen S. E. (1990). The attention system of the human brain. 13, 25–42. 2183676 10.1146/annurev.ne.13.030190.000325

[B52] Salthouse T. A. (1996). The processing-speed theory of adult age differences in cognition. Psychol. Rev. 103:403. 10.1037/0033-295X.103.3.403 8759042

[B53] Salthouse T. A. (2000). Aging and measures of processing speed. Biol. Psychol. 54, 35–54. 10.1016/S0301-0511(00)00052-1 11035219

[B54] Salthouse T. A. Atkinson T. M. Berish D. E. (2003). Executive functioning as a potential mediator of age-related cognitive decline in normal adults. J. Exp. Psychol. Gen. 132, 566–594. 10.1037/0096-3445.132.4.566 14640849

[B55] Steiger J. H. Lind J. C. (1980). Statistically-based tests for the number of common factors, in Psychometric Society's Annual Meeting (Iowa), 1–12.

[B56] Verhaeghen P. Salthouse T. A. (1997). Meta-analyses of age-cognition relations in adulthood: estimates of linear and nonlinear age effects and structural models. Psychol. Bull. 122, 231–249. 10.1037/0033-2909.122.3.231 9354147

[B57] West R. (1999). Visual distraction, working memory, and aging. Mem. Cognit. 27, 1064–1072. 10.3758/BF03201235 10586581

[B58] Wilms I. Nielsen S. (2014). Normative perceptual estimates for 91 healthy subjects age 60–75. Front. Psychol. 5:1137. 10.3389/fpsyg.2014.01137 25339932 PMC4187578

[B59] Wilson R. S. Segawa E. Boyle P. A. Bennett D. A. (2012). Influence of late-life cognitive activity on cognitive health. Neurology 78, 1123–1129. 10.1212/WNL.0b013e31824f8c03 22491864 PMC3320053

[B60] Wright S. (1921). Correlation and causation. J. Agric. Res. 20, 557–585.

